# DMF‐Based Large‐Grain Spanning Cu_2_ZnSn(S*_x_*,Se_1‐_ *_x_*)_4_ Device with a PCE of 11.76%

**DOI:** 10.1002/advs.202201241

**Published:** 2022-04-28

**Authors:** Yubo Cui, Mengyang Wang, Peizhe Dong, Shuangshuang Zhang, Junjie Fu, Libo Fan, Chaoliang Zhao, Sixin Wu, Zhi Zheng

**Affiliations:** ^1^ Key Laboratory of Micro‐Nano Materials for Energy Storage and Conversion of Henan Province Institute of Surface Micro and Nano Materials College of Chemical and Materials Engineering Xuchang University Xuchang Henan Province 461000 P. R. China; ^2^ School of Materials Science and Engineering Henan Polytechnic University Jiaozuo Henan Province 454000 P. R. China; ^3^ Key Laboratory for Special Functional Materials of Ministry of Education Henan University Kaifeng Henan Province 475004 P. R. China

**Keywords:** Cu_2_ZnSn(S*_x_*,Se_1‐_ *_x_*)_4_ (CZTSSe), dimethylformamide (DMF), large‐grain spanning, photoelectric property, redox reaction

## Abstract

A main concern of the promising DMF‐based Cu_2_ZnSn(S*_x_*,Se_1‐_ *_x_*)_4_ (CZTSSe) solar cells lies in the absence of a large‐grain spanning structure, which is a key factor for high open‐circuit voltage (*V*_oc_) and power conversion efficiency (PCE). A new strategy to achieve CZTSSe large‐grain spanning monolayer is proposed, by taking advantage of the synergistic optimization with a Cu^2+^ plus Sn^2+^ redox system and pre‐annealing temperatures. A series of structural, morphological, electrical, and photoelectric characterizations are employed to study the effects of the pre‐annealing temperatures on absorber qualities, and an optimized temperature of 430 ℃ is determined. The growth mechanism of the large‐grain spanning monolayer and the effect of redox reaction rate are carefully investigated. Three types of absorber growth mechanisms and a concept of critical temperature are proposed. The devices based on this large‐grain spanning monolayer suppress the recombination of carriers at crystal boundaries and interfaces. The champion device exhibits a high *V*_oc_ (>500 mV) and PCE of 11.76%, which are both the maximum values among DMF‐based solar cells at the current stage.

## Graphical abstract

Cu_2_ZnSn(S*_x_*,Se_1‐_ *_x_*)_4_(CZTSSe) films with large‐grain spanning structures are achieved by taking advantage of the synergistic optimization with a Cu^2+^ plus Sn^2+^ redox system and optimal pre‐annealing temperature of 430 ℃. A high open‐circuit voltage (*V*_oc_) (>500 mV) and a power conversion efficiency (PCE) of 11.76% in the champion device are both the highest values of DMF‐based CZTSSe devices at present.

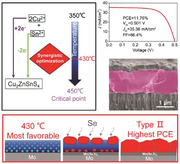

## 1 Introduction

The solution‐processed method is considered as one of the most effective methods to prepare high‐efficiency Cu_2_ZnSn(S*_x_*,Se_1‐_ *_x_*)_4_ (CZTSSe) devices.^[^ ^1^ ^]^ Mitzi group used anhydrous hydrazine to make great progress in the fabrication of high‐performance devices, but the toxic, inflammable, and explosive properties of the hydrazine limit its large‐scale commercial applications.^[^ ^2^ ^]^ Therefore, researchers explored some safe solution‐based methods.^[^ ^1^, ^3^, ^4^, ^5^, ^6^, ^7^ ^]^ The dimethyl sulfoxide (DMSO)‐based method has obtained a certified power conversion efficiency (PCE) record.^[^ ^7^ ^]^ However, DMSO‐based precursors are not stable and are required to experience heating or repeated depositional cycles to increase the solubility of the metal ions.^[^ ^8^, ^9^ ^]^ As a result, solvent engineering of Cu_2_ZnSnS_4_ (CZTS) precursors has begun to attract attention, but the role of the coordination system in it is rarely studied.^[^ ^10^, ^11^, ^12^ ^]^ Dimethylformamide (DMF) is a kind of aprotic solvent with better solubility and stability than DMSO.^[^ ^13^, ^14^ ^]^ It is reported that the solvent system of DMF and Thiourea (TU) exhibits four Lewis base sites, the O and N in DMF with the S and N in TU, which displays a unique ability to dissolve many Lewis metal compounds.^[^ ^15^ ^]^ Most importantly for Cu‐Sn‐Zn species, high solubility and stability of Cu^2+^ and Sn^2+^ in DMF and TU systems can promote the rate of the redox reaction between them to achieve the maximum. The use of DMF is beneficial to control the precursor coordination system, which overcomes the disadvantages of DMSO mentioned above.^[^ ^13^ ^]^ Therefore, it is very promising to obtain high‐efficiency CZTSSe devices by DMF.

Liu used the DMF‐based method to prepare CZTS for the first time, and obtained a low PCE of 4.77%.^[^ ^16^ ^]^ Luan et al. optimized the ratios of the cations to reach a PCE of 8.01% of CZTSSe solar cells.^[^ ^17^ ^]^ H. W. Hillhouse group controls the Ge doping concentration to fabricate CZTGSSe cells with a PCE of 11%.^[^ ^18^ ^]^ Recently, Xin et al. reported a PCE of 11.5% for the CZTSSe devices, which is the current reported top performance for DMF‐based CZTSSe devices.^[^ ^19^ ^]^ The use of Sn^4+^ in precursor solution in the annealing process directly produces CZTS, which effectively avoids the production of other sulfides, including ZnS, SnS and reduces the production of the secondary phase (ZnSe, SnSe_2_). However, double‐layer cross‐sectional morphology is produced by this method, limiting the device efficiency. Research effort shows that the double or triple layer leads to increased grain boundaries, increased recombination centers,^[^ ^20^, ^21^, ^22^, ^23^ ^]^ high open‐circuit voltage (*V*_oc_) loss, which undermine the device efficiency.^[^ ^24^, ^25^, ^26^ ^]^

It is very commonly accepted that CZTSSe morphology can be controlled during the fabrication process of the absorber in a DMF‐based device. There are mainly the following three methods to realize the control of the CZTSSe morphology. The first method is alkali metal doping. Liu et al. utilized Na element doping to increase the compactness and decrease the voids of the absorbers, leading to increased efficiency from 4.77% to 5.8%.^[^ ^16^ ^]^ The second method is element ratio control. Luan et al. control the ratio of Cu:(Zn+Sn) and Zn:Sn to decrease the voids of cross‐section and obtain compact CZTSSe absorbers.^[^ ^17^ ^]^ The third method is to optimize the precursor reaction process. Xin et al. used Sn^4+^and Cu^1+^ as the starting materials to decrease the redox reaction process in the fabrication of CZTSSe absorbers and avoid secondary phase production, leading to the production of compact and void‐free absorbers.^[^ ^19^ ^]^ Large‐grain spanning monolayer is vital to obtain high‐efficiency devices. However, it is still a challenge to obtain this structure by the DMF‐based method even though large‐grain spanning absorbers can be obtained by this strategy. This is the bottleneck of the DMF‐based method.

A new DMF‐based solution‐processed method was developed in this paper, where Cu(oAC)_2_·H_2_O, SnCl_2_·2H_2_O, ZnCl_2_, TU were employed as starting materials to achieve large grains at the upper contact interface and small grains on the back contact interface, which will form a typical large‐grain spanning monolayer prepared at the optimized pre‐annealing temperature of 430 ℃. In the process of selenization, this kind of cross‐sectional structure can help large grains under the upper contact interface merge with the small grains on the back contact interface, leading to the formation of a large‐grain spanning monolayer, which is compact, void‐free, and perfect contact with Mo substrates. This strategy, for the first time, realizes the controllable formation of large‐grain spanning monolayer prepared by the DMF‐based method. The redox reaction of Sn^2+^and Cu^2+^, namely Sn^2+^ is oxidized to Sn^4+^ , Cu^2+^ is reduced to Cu^1+^, happened. This intermediate reaction process stabilizes the final states of Cu and Sn in CZTSSe and effectively suppresses the secondary phases of CuS(Se) and SnS(Se). By the synergetic optimization with the redox reaction of Sn^2+^ and Cu^2+^ and pre‐annealing temperatures, large‐grain spanning absorbers can significantly improve the charge concentration and suppress non‐radiative recombination of the carriers, leading to improved shunt resistance (*R*_sh_) and recombination resistance (*R*_ct_) of CZTSSe cell. An enhanced *V*_oc_ of 501 mV and a high PCE of 11.76% are obtained, which is the highest value in CZTSSe solar cells prepared by the DMF‐based method at present.

## 2 Results and Discussions

**Figure 1:**
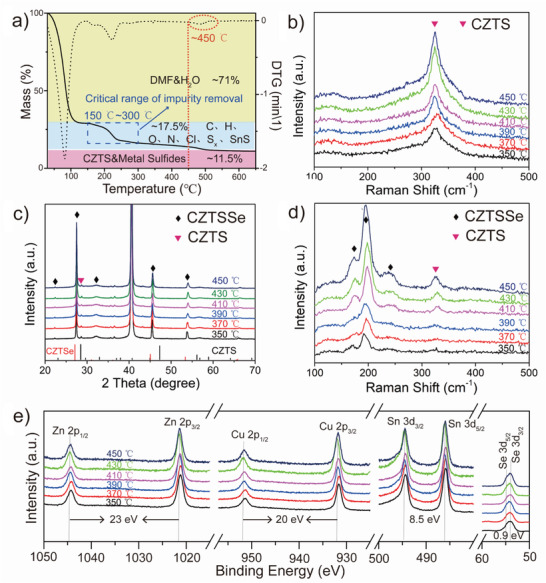
a) TGA curves of the precursor solution. b) Raman spectra of CZTS films. c) XRD patterns of CZTSSe films. d) Raman spectra of CZTSSe films. e) XPS spectra of CZTSSe films.

The pre‐annealing temperature is the key parameter of the new DMF‐based solution‐processed method, which can greatly influence the crystallinity of absorbers and the rate of redox reaction between Cu^2+^ and Sn^2+^. Thermal Gravimetric Analysis (TGA) was utilized to determine the range of the pre‐annealing temperature (**Figure** **1**a). The mass loss before 150 ℃ is attributed to the volatilization of the DMF and H_2_O.^[^ ^27^ ^]^ The mass loss between 150 and 550 ℃ is ascribed to the degradation of the metal salts, the volatilization of the complex polymers (metal salt‐DMF complex), and the loss of the sulfur and tin sources.^[^ ^27^, ^28^ ^]^ The dominated mass loss is in the region of 150–300 ℃, which indicates the volatile components are the main mass loss in the precursors. The mass keeps invariable as the temperature is beyond 550 ℃, indicating that the residual substance should be CZTS and other chalcogenides.^[^ ^27^ ^]^ Different mass ratios of the precursor are shown in Figure S1 (Supporting Information). Pre‐annealing temperature is chosen to be 350–450 ℃ to get rid of the complex polymers in the precursor and assure the safety of the experiments within the glovebox. A mass loss ratio peak at 450 ℃ is observed. This temperature shows the critical value of the significantly increased redox reaction rate, which will be discussed in Figure 4.

To evaluate the influence of pre‐annealing temperatures on the crystallinity of the CZTS precursor films, Raman measurements were performed (Figure 1b). It is observed that the intensity of the peak at 326 cm^–1^, which is the characterization peak of CZTS,^[^ ^29^ ^]^ gradually increases and becomes sharp as the pre‐annealing temperature increases from 350 to 450 ℃, indicating the increased crystallinity. This result is also confirmed by the XRD patterns shown in Figure S2 (Supporting Information).

The X‐ray diffraction (XRD) patterns of the CZTSSe absorbers could be attributed to Cu_2_ZnSnSe_4_ (CZTSe, PDF NO.52‐0868) and CZTS (PDF NO.26‐0575), the peak at 40.5°is attributed to the Mo substrates (Figure 1c). The weak diffraction peaks at 32.2° are attributed to Mo(Se,S)_2_.^[^ ^19^ ^]^ No additional peaks from impurity are observed. The full width at half maximum values of (112) peak of the CZTSSe decreases from 0.23° to 0.07° with the increase in temperature, which indicates that the crystallinity of the absorbers is improved after pre‐annealing (Figure S3, Supporting Information). In addition, *I*_(112)_/*I*_(204)_ increases with the increased pre‐annealing temperature, which suggests the increased preferential orientation of (112) plane (Table S1, Supporting Information).

To further confirm the phase purity of the CZTSSe films, Raman spectra were collected (Figure 1d). The peaks at 173.5, 195.7, and 239.2 cm^–1^ are attributed to the characteristic peaks of CZTSSe. The peak at 326 cm^–1^ is the characteristic peak of CZTS.^[^ ^19^ ^]^ There are no additional secondary phases. Meanwhile, CZTSSe Raman peaks become sharp as the temperature increases from 350 to 450 ℃, indicating the increased crystallinity caused by the pre‐annealing process, which is consistent with the XRD results. It is noted that both XRD and Raman results show that the precursor films and the absorbers do not have secondary phases. Niu et al.^[^ ^19^ ^]^ used Cu^1+^ and Sn^4+^ as reaction materials to effectively avoid the production of the secondary phases. However, Cu^2+^ and Sn^2+^ were employed to ensure the high purity of CZTSSe in this work. Cu(oAC)_2_·H_2_O, ZnCl_2_, and TU form two kinds of complexes (see Equation 1),^[^ ^19^ ^]^ SnCl_2_·2H_2_O and DMF from the third kind of complex (see Equation 2).^[^ ^19^ ^]^ Redox reaction occurs among the above three kinds of complexes. Cu^2+^ is reduced to Cu^1+^ and Sn^2+^ is oxidized to Sn^4+^, leading to the CZTS production (see Equation 3). This redox reaction stabilizes the states of the elements, avoiding the secondary phase generation, which provides prerequisites to obtain a large‐grain spanning monolayer structure. This result will be discussed in detail. The reaction of the selenization process of the precursor is shown in Equation 4.

(1) $$\mathrm{Cu}{\left(\mathrm{oAC}\right)}_{2}+Z\mathrm{nC}l_{2}+\mathrm{TU}\to \mathrm{Cu}{\left(\mathrm{Tu}\right)}_{x}{\left(\mathrm{oAC}\right)}_{2}+\mathrm{Zn}{\left(\mathrm{TU}\right)}_{x}Cl_{2}$$

(2) $$\mathrm{SnC}l_{2}+\mathrm{DMF}\to \mathrm{Sn}{\left(\mathrm{DMF}\right)}_{x}Cl_{2}$$

(3) $$\begin{array}{c} 2\mathrm{Cu}{\left(\mathrm{TU}\right)}_{x}({\mathrm{oAC})}_{2}+\mathrm{Sn}{\left(\mathrm{DMF}\right)}_{x}Cl_{2}+\mathrm{Zn}{\left(\mathrm{TU}\right)}_{x}Cl_{2}\to Cu_{2}\mathrm{ZnSn}S_{4} \end{array}$$

(4) $$Cu_{2}\mathrm{ZnSn}S_{4}+4\mathrm{Se}\to Cu_{2}\mathrm{ZnSn}{\left(S,\mathrm{Se}\right)}_{4}$$

To confirm the valence states of CZTSSe films, X‐ray photoelectron spectroscopy (XPS) was performed (Figure 1e). The XPS of Cu exhibits 2p two peaks with binding energies of 952.1 eV (2p_1/2_) and 932 eV (2P_3/2_). The separation of the two peaks is ≈20 eV, which is consistent with Cu^1+^. The peaks with the binding energies of 1044.8 and 1021.8 eV show the separation energy of 23 eV, which is attributed to the Zn^2+^. Sn^4+^ shows two peaks at 494.7 and 484.2 eV with 8.5 eV separation energy. Se 3d peaks are located at 54.8 and 53.9 eV with 0.9 eV separation binding energy. XPS results show that all the elements are in the expected valence states.^[^ ^14^, ^30^ ^]^ In addition, all the characterization peaks of the metal ions shift toward the high‐energy side. The electronegativity of S is higher than that of Se, and the Se amount decreases in the final product as the pre‐annealing temperature increases from 350 to 450 ℃, leading to the shift toward the high‐energy side.^[^ ^6^ ^]^ This result is consistent with the shift of (112) planes toward the large‐angle side in XRD data (Figure S4, Supporting Information).

**Figure 2:**
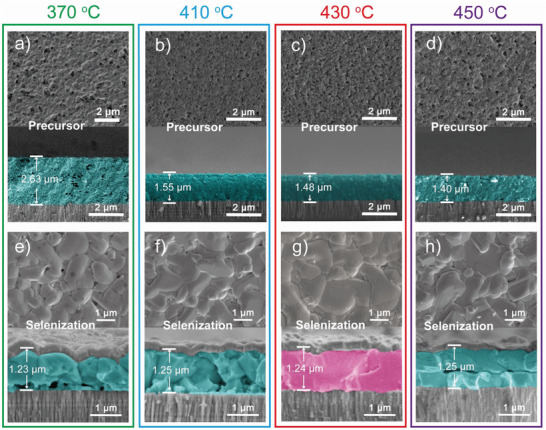
Top‐view and cross‐sectional images of a–d) CZTS precursors and e–h) CZTSSe absorbers at the pre‐annealing temperature of 370, 410, 430, and 450 ℃.

Large‐grain spanning morphology of the CZTSSe absorber is observed in scanning electron microscopy (SEM) images at the pre‐annealing temperature of 430 ℃. Morphology of the CZTS films before and after selenization was collected. To study the influence of the pre‐annealing temperature, top‐view and cross‐sectional SEM images of the CZTS films are shown in **Figure** **2**a–d. The surface and cross‐section of the films exhibit a lot of holes and voids, and the thickness is about 2.63 µm as the temperature is 370 ℃ (Figure 2a). When the temperature increases to 410 ℃ (Figure 2b), the holes at the surface and voids at the cross‐section decrease. Meanwhile, the thickness is ≈1.55 µm. As the temperature reaches 430 ℃ (Figure 2c), the film thickness of 1.48 µm does not change that much, but the surface and cross‐sectional morphology become more compact, which benefits the following selenization process. As the temperature increases to 450 ℃ (Figure 2d), the film thickness decreases to 1.40 µm, which is possibly attributed to the evaporation of complex polymers caused by increased temperature. Based on the compactness of the films, combining the XRD and Raman results, 430 ℃ is the optimized pre‐annealing temperature. The morphology of absorbers prepared under other pre‐annealing temperatures is shown in Figure S5 (Supporting Information).

Figure 2e–h shows the top‐view and cross‐sectional SEM images of the CZTSSe absorbers obtained by precursor films after selenization. Thicknesses of the films at 350, 370, 390, 410, 430, and 450 °C are 1.28, 1.23, 1.27, 1.25, 1.24, and 1.25 µm, respectively (Figure S6, Supporting Information). As the pre‐annealing temperature is 370 ℃ (Figure 2e), obvious holes are shown on the surface, a big‐grain layer can be seen under the upper contact interface, and on the back contact interface of the cross section shows a small‐grain layer with bad compactness. There are a lot of holes among grains. The holes at the surface and voids at the cross‐section decrease as the temperature increases from 370 to 410 ℃ (Figure 2f). A small‐grain layer is still shown on the back contact interface of the cross‐section, but the boundary between the big‐grain layer under the upper contact interface and the small‐grain layer on the back contact interface becomes blurred. As the temperature increases to 430 ℃ (Figure 2g), large grains and flat surfaces are shown. The cross‐section shows the compact large‐grain spanning monolayer connected tightly with the Mo substrate. As the temperature increases to 450 ℃ (Figure 2h), fewer holes are observed. Considering the data in Figure 2d, the holes at the surface and voids at the cross‐section may be one of the possible reasons for this result. Both large‐grain layers under upper and on back contact interfaces, respectively, with obvious boundary can be seen. SEM images of other absorber films after selenization are shown in Figure S6 (Supporting Information).

**Figure 3:**
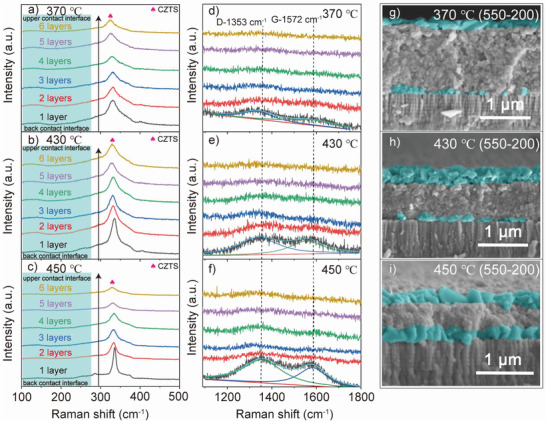
Raman spectra of the CZTS precursors spin‐coated 1–6 layers in the range of a–c) 100–500 cm^–1^ and d–f) 1200–1800 cm^–1^. g–i) Cross‐sectional SEM images of the precursors with six layers after the early stage of selenization at pre‐annealing temperatures of 370, 430, and 450 °C.

Briefly, compact precursor films are obtained by the pre‐annealing temperature of 430 ℃, and absorbers with a cross‐sectional morphology of large‐grain spanning monolayer are obtained after selenization. Thus, three types of cross‐sectional morphologies are observed from different pre‐annealing temperatures. The first type of morphology (Type I, 350–410 ℃) exhibits a small‐grain layer on the back contact interface and a big‐grain layer under the upper contact interface. The second kind of morphology (Type II, 430 ℃) shows a large‐grain spanning monolayer. The third kind of morphology (Type III, 450 ℃) is both large‐grain layers under upper and on back contact interfaces, respectively, with obvious boundaries. To explain the growth mechanism of the large‐grain spanning monolayer, **Figure** **3** shows Raman and SEM results of the precursor films prepared by different spin‐coating layers.

The preparation of large‐grain spanning absorbers is significant to fabricate devices with high efficiency. To realize controllable preparation, the growth mechanism is investigated. Raman and SEM were performed on the CZTS films prepared under three representative pre‐annealing temperatures (370, 430, and 450 ℃). On this basis, a synergistic optimization with redox reaction and the pre‐annealing temperature was proposed.

By preparing samples with different spin‐coating layers, Raman spectra of CZTS films were conducted. The preparation process is shown in Figure S7 (Supporting Information). Early‐stage of selenization (550 ℃, 200 s) was performed to observe the crystal growth of the cross‐section. The Raman and SEM results are shown in Figure 3.

When the pre‐annealing temperature is 370 ℃ (Figure 3a), 430 ℃ (Figure 3b), and 450 ℃ (Figure 3c), the CZTS characteristic peak of the precursor, from back contact interface to upper contact interface, gradually weakens with the increased layers. For the same number of layers, the crystallinity gradually increases with the increase of temperature. The crystallinity of the precursor with only one layer at three temperatures is displayed in Figure S8 (Supporting Information). This indicates that CZTS are inclined to crystallize on the back contact interface during pre‐annealing. Increasing the temperature can improve the crystallinity of CZTS film; the critical temperature reaches 450 ℃, at which the redox reaction rate is significantly increased, as described in Formula 3, which is consistent with the above TGA results.

Two peaks at 1353 and 1572 cm^–1^ are the D and G Raman scattering peaks corresponding to a graphitic structure, which is caused by the decomposition of the complex polymers, as shown in Figure 3d–f.^[^ ^27^ ^]^ The graphitized degree of complex polymers in precursor decreases from the back contact interface to the upper contact interface at 370 ℃ (Figure 3d), 430 ℃ (Figure 3e), and 450 ℃ (Figure 3f), which is similar to the results of CZTS films discussed above. The more graphitic structure is located at the back contact interface, which is due to the longer pre‐annealing time of the precursor near this interface, resulting in more graphitization of the complex polymers. The graphitized degree of the precursor with only one‐layer increases with the rising temperature, as shown in Figure S9 (Supporting Information). Note that this graphitic structure is favorable to the crystal growth of CZTSSe.^[^ ^12^, ^27^, ^31^ ^]^

As the pre‐annealing temperature increases, the residual complex polymers in the precursor film decrease.^[^ ^29^, ^32^ ^]^ Complex polymers tend to hinder the diffusion of selenium vapor, so reducing the complex polymers residue will promote crystal growth during selenization. The higher the temperature, the better crystallinity of the precursor, and the larger grain size at the upper contact interface during selenization. At 370 ℃, small grains of ≈100 nm grow on the back contact interfaces of absorbers (Figure 3g). At 430 ℃, the grain size increases slightly (Figure 3h), while the grain size increases significantly to 200–300 nm, and the grain amount increases meanwhile at 450 ℃ (Figure 3i). A similar phenomenon has been observed under the upper contact interface of the absorber. The grain size is obviously bigger both at 430 and 450 °C than those at 370 °C. The grain sizes on the back and under the upper contact interfaces are smaller at 370 ℃ than those at 430 and 450 °C.

In the late selenization stage, it is easy to form a cross‐sectional morphology of upper contact interface with big grains and back contact interfaces with small grains and many voids at 370 ℃, named Type I. At 430 ℃, big grains grow under the upper contact interface, and small grains grow on the back contact interface, which is easy to form a large‐grain spanning monolayer called Type II. At 450 ℃, grain size is large under the upper contact interface, which is similar to that on the back contact interface; thus, the crystals compete with each other and divide into two layers at the later growth stage, which is not conducive to the formation of large‐grain spanning monolayer, named Type III.

**Figure 4:**
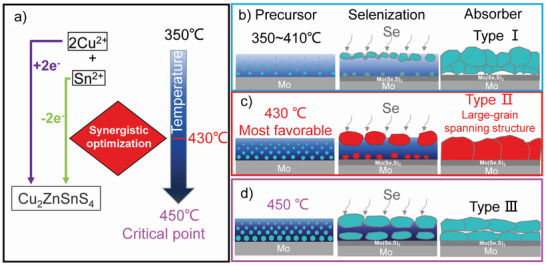
a) Schematic of synergistic optimization with redox reaction and pre‐annealing temperature. b) Growth modes of precursor, selenization, and absorber at 350–410 ℃, c) at most favorable 430 ℃, and d) at critical point 450 ℃.

A schematic of synergistic optimization with redox reaction and pre‐annealing temperature is shown in **Figure** **4**a. It can be seen that 2 mol of Cu^2+^ is reduced to Cu^1+^, gaining 2 mol electrons, and 1 mol of Sn^2+^ is oxidized to Sn^4+^, losing 2 mol electrons, resulting in CZTSSe. The so‐called synergistic optimization with redox reaction and pre‐annealing temperature means that the rate of redox reaction gradually becomes fast as the pre‐annealing temperature increases from 350 ℃ to the critical temperature of 450 ℃. Low reaction rates lead to small‐grain layers with many voids after selenization on the Mo interface (350–410 ℃). When the reaction rate is appropriate, the amount of CZTS on the back contact interface is small. At the same time, this temperature also ensures the removal of impurity polymers and promotes the formation of large grains under the upper contact interface in the selenization process. In the late stage of selenization, the large grains under the upper contact interface can merge with the small grains on the back contact interface to form a large‐grain spanning monolayer (430 ℃). When the temperature increases to the critical temperature (450 ℃), the reaction rate also increases significantly. In the selenization stage, an excessive reaction rate will generate too many crystal nuclei on the back contact interface, which will prevent the large grains under the upper contact interface from absorbing the grains on the back contact interface and result in a double‐layer structure.

As shown in Figure 4b, when the pre‐annealing temperature is in the range of 350–410 ℃, there are more organic impurities in the precursor film and relatively few CZTS grains with poor crystallinity. During the process of selenization, smaller CZTS crystal nuclei are unstable and inclined to decompose, while stable ones grow bigger. The absorber shows a morphology with smaller grain size and more grain boundaries accompanied by the existence of voids (Type I). As the temperature increases to 430 ℃ (Figure 4c), the residual polymers in the precursor film gradually decrease. During the selenization process, the crystal growth rate increases. And the grain growth rate at the upper interface is significantly greater than that at the back contact interface. The larger grains under the upper contact interface merge with the smaller grains on the back contact interface to form a large‐grain spanning monolayer (Type II). As the pre‐annealing temperature rises to 450 ℃ (Figure 4c), the grains of the absorber near the back interface during the selenization process are quite larger as compared with 370 and 430 ℃, and the grains near the upper interface cannot merge with the larger grains of the back contact interface. A double‐layer structure formed (Type III).

The synergistic optimization of the pre‐annealing temperature and the redox reactions of Sn^2+^ and Cu^2+^ can obtain a precursor film with the most suitable amount of grains, crystallinity, and degree of graphitization, which also has a direct effect on the process of selenization crystal growth. Temperature adjustment can effectively control the amount of crystals and crystallinity at the back interface to be in an optimal range, so that the large grains at the upper interface can merge with the small grains at the back interface, which can obtain a large‐grain spanning structure in a controllable manner.

In brief, we report a controllable preparation method that can effectively obtain a large‐grain spanning monolayer, which is derived from the synergistic optimization with the redox reaction of Cu^2+^ and Sn^2+^ and pre‐annealing temperature. Obviously, this large‐grain spanning absorber is conductive to fabricate high‐efficiency CZTSSe devices.

**Figure 5:**
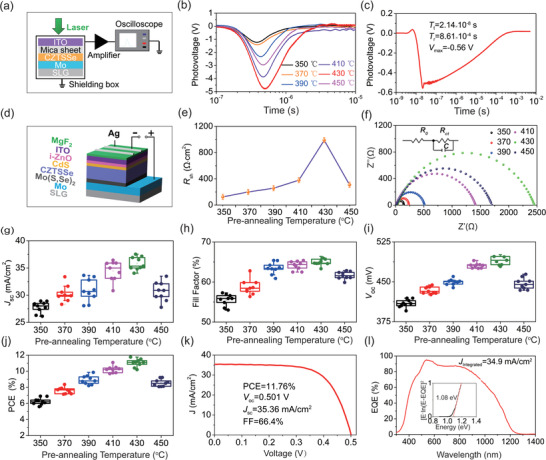
a) TSPV test configuration. b) TSPV curves of the CZTSSe films prepared with different pre‐annealing temperatures. c) TSPV curve of the champion device. d) Schematic of CZTSSe device. e) The relationship between *R*_sh_ and pre‐annealing temperature. f) Nyquist plot of the devices based on different annealing temperatures. g) Average PCE. h) Average *J*_sc_, i) average *V*_oc_, j) average FF, k) *J*–*V* curve of the champion device. l) EQE curve of the champion device, the inset shows the bandgap obtained by fitting.

To predict the photovoltaic performance of devices assembled with large‐grain spanning absorbers, a transient surface photovoltage (TSPV) test was conducted on CZTSSe films.^[^ ^33^, ^34^, ^35^, ^36^ ^]^ TSPV test configuration is illustrated in **Figure** **5**a. A 355 nm pulse laser with a pulse width of 4 ns was first processed by a reflector and then utilized for exciting the CZTSSe films in a shielding box. The signals were recorded by a digital TDS oscilloscope. The laser was excited on the top of the sample covered by a layer of mica spacers, which was sandwiched between ITO and Mo electrodes.

It can be seen from Figure 5b that all the absorbers prepared by different pre‐annealing temperatures show negative TSPV signals, which indicates that the light‐induced holes accumulate on the Mo electrode. Therefore, all the samples are P‐type semiconductors.^[^ ^37^, ^38^ ^]^ As the temperature increases from 350 to 430 ℃, the maximum of the photovoltage (*V*_max_), the separation time of the carriers (*T*_t_, time at the peak of TSPV curves), and the recombination time of the carriers (*T*_r_, time at the beginning of the platform of TSPV curves) increase from 1.24 V, 3.81 × 10^–7^  s and 1.12 × 10^–6^  s to 4.81 V, 5.02 × 10^–7^ s and 2.89 × 10^–6^ s, separately (**Table** **1**). In comparison, the increasing degree of *T*_r_ is bigger than that of *T*_t_, which is beneficial to increase the lifetime of carrier transporting at the interface. The absorber prepared at 430 ℃ has the highest concentration and the longest lifetime of the carrier at the interface. In addition, the smaller the value of *T*_t_/*T*_r_ is, the faster the speed of carriers in the absorber is. By calculation, the value of *T*_t_/*T*_r_ is 0.17 at 430 ℃, which is the smallest one among all the values at different temperatures. This illustrates the absorber pre‐annealed at this temperature exhibits the highest quality because of the formation of a large‐grain spanning monolayer, which can obviously reduce the recombination of carriers at the interface and grain boundary. Figure 5c is the TSPV curve of the champion device prepared by the best absorber of 430 ℃. The test was conducted without an amplifier, and its configuration can be seen in Figure S10 (Supporting Information). The *V*_max_, *T*_t_, and *T*_r_ are 0.56 V, 2.14 × 10^–8^ s, and 8.61 × 10^–4^ s, respectively.

**Table 1:** The TSPV parameters of the CZTSSe films prepared at different pre‐annealing temperatures

Pre‐annealing tempurature (℃)	*V*_max_ (V)	*T*_t_ (s)	*T*_r_ (s)	*T*_t_/ *T*_r_
350	1.24	3.81 × 10^–7^	1.12 × 10^–6^	0.34
370	1.43	3.92 × 10^–7^	1.23 × 10^–6^	0.32
390	2.36	4.35 × 10^–7^	1.48 × 10^–6^	0.29
410	3.90	4.71 × 10^–7^	2.21 × 10^–6^	0.21
430	4.81	5.02 × 10^–7^	2.89 × 10^–6^	0.17
450	3.00	4.71 × 10^–7^	1.68 × 10^–6^	0.28

The schematic of CZTSSe solar cells is shown in Figure 5d, which is illustrated detailly in the Experimental Section. As known, the voids in the absorber will create new shunt paths in the devices, which has a significant impact on the *R*_sh_ of the devices.^[^ ^20^, ^39^ ^]^ The *R*_sh_ of the devices prepared at different pre‐annealing temperatures was obtained by fitting current density–voltage (*J*–*V*) curves, as shown in Figure 5e. The fitting method is shown in Figure S12 (Supporting Information). As the temperature increases from 350 to 430 ℃, the *R*_sh_ increases from 125 Ω cm^[^ ^2^ ^]^ to a maximum of 990 Ω cm^[^ ^2^ ^]^ and then decreases to 309 Ω cm^[^ ^2^ ^]^ when the temperature further increases to 450 ℃, which can be attributed to the formation of large‐grain spanning monolayer at 430 ℃. Similar results have also been observed in electrochemical impedance spectroscopy (EIS). Figure 5f is the Nyquist plot of the device prepared by different pre‐annealing temperatures. The diameter of the curve is related to the *R*_ct_ of the device.^[^ ^40^ ^]^ As the temperature rises from 350 to 430 ℃, the *R*_ct_ firstly increases from 140 Ω to the maximum of 2433 Ω and then decreases to 1697 Ω when the temperature increases continually to 450 ℃.

It is similar to *R*_sh_ and *R*_ct_, the average values of short‐circuit current density (*J*_sc_), fill factor (FF), *V*_oc,_ and PCE increase from 27.70 mA cm^−2^, 55.52%, 408 mV, 6.21% to the maximum of 35.83 mA cm^−2^, 65.03%, 491 mV and 11.04% as the pre‐annealing temperature increasing from 350 to 430 ℃, and decrease to 30.66 mA cm^−2^, 61.66%, 447 mV and 8.56% when the temperature increased further to 450 ℃, as shown in Figure 5g–j. Obviously, the devices fabricated by the absorber pre‐annealed at 430 ℃ exhibited the optimum photoelectric properties. The performance parameters of the champion device obtained at different pre‐annealing temperatures are shown in Table S2 ( Supporting Information). At present, the highest PCE of 11.76% and *V*_oc_ of 501 mV in DMF‐based CZTSSe devices is displayed in the *J*–*V* curve of the champion device fabricated under the pre‐annealing condition of 430 ℃ (Figure 5k). *V*_oc, deficit_ can be calculated with *V*_oc, deficit_ = *E*_g_ − *V*_oc_, according to the research of Haass.^[^ ^41^ ^]^ Therefore, *V*_oc, deficit_ of our work is 0.579 V(*V*_oc, deficit_ = 1.08 − 0.501), which is lower by 0.01 V when compared with 0.589 V of Xin's work (*V*_oc_, _deficit_ = 1.08 − 0.491).^[^ ^19^ ^]^ Meanwhile, *J*_sc_, and the FF are 35.36 mA cm^−2^ and 66.4%, respectively. The *J*‐*V* curves of the device prepared at other temperatures conditions are shown in Figure S12 (Supporting Information). Figure 5l displays the corresponding external quantum efficiency (*EQE*) curve of the champion device. The current density calculated by the *EQE* curve is 34.9 mA cm^−2^ close to the experimental value, and the bandgap of the device is fitted to be 1.08 eV (insert in Figure 5l). The *EQE* curves of other samples at different temperatures can be seen in Figure S13 (Supporting Information).

The formation of a large‐grain spanning structure greatly reduces grain boundaries, which can significantly reduce carrier recombination. Comparing devices at different temperatures, the FF of the device at 430 °C is significantly higher than other counterparts. Therefore, the *R*_sh_ at 430 °C is increased by nearly five times compared with that at 370 °C. On the other hand, the *R*_s_ at different temperatures are all between 0.35 Ω and 0.53 Ω, which fluctuates relatively little. This phenomenon shows that different temperatures have relatively little effect on charge transportation. According to the above analysis, the significant improvement of FF and *R*_sh_ brought about by the formation of the large‐grain spanning structure provides a major role in improving the device efficiency, while the *R*_s_ value has a relatively minor impact.

In Xin's work, Sn^4+^ and Cu^1+^ are used as raw materials to avoid the generation of secondary phases in the absorbers by reducing the redox process. Similarly, there are no secondary phases observed in absorbers prepared with starting materials of Cu^2+^ plus Sn^2+^ in our work. More importantly, a large‐grain spanning structure can be obtained at the most favorable redox reaction rate and mount of grains through the synergistic optimization of the Cu^2+^ plus Sn^2+^ redox process and the 430 ℃ pre‐annealing temperature. The degree of graphitization of impurity polymer in the precursor film is moderate, which provides favorable conditions for the crystal growth stage, so that large grains are formed at the upper interface during the selenization process, which can merge the small grains at the Mo interface, thereby forming a flat surface and a dense cross section. The large‐grain spanning structure, highly desirable in CZTSSe solar cells, can reduce the grain boundaries in the absorbers and suppress carrier recombination. All in all, we believe that the synergistic optimization effect of this work is a controllable and effective path to improve the performance of CZTSSe devices.

Our research results are expected to be applied to solution systems employing metal salts as solutes by using Cu^2+^ and Sn^2+^, such as sulfides, chlorides, etc. By adjusting the pre‐annealing temperature and redox reaction rate of the precursor solution, an appropriate precursor film can be obtained, and the absorption layer in the selenization stage can be promoted to grow into a monolayer large‐grain spanning structure.

## 3 Conclusion

In this paper, DMF‐based CZTSSe solar cells were prepared with a PCE of 11.76%, which is the highest value of devices in this solution system at present. It was also found that the synergistic optimization with the redox reaction of Cu^2+^ and Sn^2+^ and the pre‐annealing temperature was a controllable preparation method that could effectively obtain a large‐grain spanning monolayer within the absorber. The redox reaction of Cu^2+^ and Sn^2+^ is affected by the pre‐annealing temperature, resulting in different reaction rates and crystal amounts at the Mo interface. The results show that the pre‐annealing temperature of 430 ℃ is optimum, which promotes the formation of large grains at the upper contact interface during selenization, and small grains at the Mo interface can be merged. Thus, forming a large‐grain spanning monolayer with a flat surface and dense cross‐section, which can inhibit the recombination of carriers at the interface and grain boundary within the absorber and significantly improve the *R*_sh_ and *R*_ct_ values of the device. *J*_sc_ of 35.36 mA cm^–2^ and FF of 66.4% are also the largest results obtained from the device prepared at this temperature among all the samples. In particular, *V*_oc_ of the device reached 501 mV, which is the optimal value for DMF‐based devices. The results of this study provide a way to effectively improve the efficiency of CZTSSe devices, namely the controllable preparation of large‐grain spanning monolayer and provide a theoretical basis for the development of DMF‐based devices.

## 4 Experimental Section

### Chemicals

Copper acetate monohydrate (Cu(oAC)_2_·H_2_O, 99.95%), Cadmium sulfate (CdSO_4_·8/3H_2_O, 99.99%), and selenium (99.999%) were purchased from Shanghai Aladdin Biochemical Technology Co., Ltd. Zinc chloride (ZnCl_2_, 99.95%), Tin(II) chloride dihydrate (SnCl_2_·2H_2_O, 98%) were purchased from Thermal Fisher Scientific. Thiourea (C(NH_2_)_2_S, analytically pure reagent), Ethanol (C_2_H_5_OH, analytically pure reagent), 2‐propanol ((CH_3_)_2_CHOH, analytically pure reagent), Ammonia (NH_3_·H_2_O, analytically pure reagent) were purchased from Sinopharm Chemical Reagent Co., Ltd. *N,N‐*dimethylformamide (DMF, 99.9%) was purchased from  Beijing Innochem Science & Technology Co., Ltd. Acetone (CH_3_COCH_3_, 99.9%) was purchased from Tianjin Fuyu Fine Chemical Co., Ltd.

### Preparation of the Precursor Solution

The precursor solution was prepared in the air. All chemicals except thiourea were used as received without further purification. Thiourea was recrystallized once with ethanol. 0.5420 g Cu(oAC)_2_·H_2_O, 0.3700g SnCl_2_·2H_2_O, 0.3060 g ZnCl_2_, and 1.063 g thiourea were weighted and kept in a vial. Then 5 mL DMF was injected, and the solution was kept stirring for 3 h (1000 rpm) to obtain a light‐yellow solution.

### Fabrication of CZTSSe Absorber Films

The precursor films were prepared in a glove box filled with argon gas and H_2_O and O_2_ level below 10 ppm. The precursor solution was spin‐coated on the molybdenum‐coated soda‐lime glass (MSLG) substrate at 800 rpm for 5 s and 3000 rpm for 30 s, and then annealed on the hot plate at different temperatures for 1.5 min to prepare the precursor films. MSLG substrates were sequentially cleaned by acetone and 2‐propanol under sonication for 20 min each and dried under 60 ℃ in the air. The precursor solution was placed on a hot plate to keep at 45 ℃ and filtered with a 0.22 µm PTFE filter before spin‐coating. Coating‐annealing‐cooling cycle and cooling time was 7.5 min, which was repeated six times to obtain the precursor film. The precursor films were put into a graphite box with Se granules (≈1.0 g) and annealed in a rapid thermal processing (RTP) furnace (so‐called selenization) to form the absorber film with an Ar flow rate of 80 sccm. The furnace was heated to 550 °C from room temperature within 1 min and held at this temperature for 15 min, and the furnace was cooled down naturally.

### Fabrication of CZTSSe Solar Cells

To complete the fabrication of the devices, a CdS buffer layer (≈50 nm) and i‐ZnO (≈70 nm)/ITO (≈200 nm) bilayers were successively deposited by chemical bath deposition and RF magnetron sputtering, respectively. A silver gate electrode was deposited by thermal evaporation on the top of the devices. Finally, an MgF_2_ anti‐reflection layer with a thickness of 80 nm was deposited by thermal evaporation on the CZTSSe solar cell that had an area of 0.21 cm^2^.

### Characterization

TGA was performed on a METTLER TOLEDO TGA2 instrument in an alumina crucible under a flowing argon atmosphere with a heating rate of 5 ℃ min^−1^. The crystal structures were characterized by an X‐ray powder diffractometer (Bruker D8‐Advanced) with a Cu K*α* (*λ* = 1.54056 Å, 40 kV, 40 mA) source with a step size of 0.02°. Raman spectra were recorded on a Renishaw Raman microscope (inVia, Renishaw, UK) using an excitation laser with 532 nm wavelength. Morphologies of samples were studied by SEM (Nova NanoSEM 50) at an accelerating voltage of 15 kV. XPS was performed using a Thermo Fisher Scientific K‐alpha X‐ray photoelectron spectrometer. To conduct TSPV, A 355 nm pulsed laser was used as the light source to excite the CZTSSe films, and the voltage signals were recorded by an oscilloscope (Tektronix TDS 3054C, 500 MHz). The EIS was performed on a frequency response analysis (FRA) equipped PGSTAT‐30 from Autolab (AUT302N, Metrohm, Switzerland), scanning from 6 MHz to 50 Hz under −0.4 V. *J*–*V* curves were tested on a Xe light source‐based solar simulator, and the light intensity was calibrated to AM 1.5 (100 mW cm^–2^) using a standard Si reference cell. The *EQE* was performed using a Zolix SCS100 QE system installed with a 150 W xenon light source and a lock‐in amplifier.

## Conflict of Interest

The authors declare no conflict of interest.

## Supporting information

Supporting Information

## Data Availability

Research data are not shared.

## References

[1] T. Todorov , H. W. Hillhouse , S. Aazou , Z. Sekkat , O. Vigil‐Galán , S. D. Deshmukh , R. Agrawal , S. Bourdais , M. Valdés , P. Arnou , D. B. Mitzi , P. J. Dale , J. Phys. Energy 2020, 2, 012003.

[2] W. Wang , M. T. Winkler , O. Gunawan , T. Gokmen , T. K. Todorov , Y. Zhu , D. B. Mitzi , Adv. Energy Mater. 2014, 4, 1301465.

[3] Z. Su , J. M. R. Tan , X. Li , X. Zeng , S. K. Batabyal , L. H. Wong , Adv. Energy Mater. 2015, 5, 1500682.

[4] A. R. Uhl , C. Fella , A. Chirilă , M. R. Kaelin , L. Karvonen , A. Weidenkaff , C. N. Borca , D. Grolimund , Y. E. Romanyuk , A. N. Tiwari , Prog. Photovolt.: Res. Appl. 2012, 20, 526.

[5] H.‐Q. Xiao , W.‐H. Zhou , D.‐X. Kou , Z.‐J. Zhou , Y.‐N. Meng , Y.‐F. Qi , S.‐J. Yuan , Q.‐W. Tian , S.‐X. Wu , Green Chem. 2020, 22, 3597.

[6] A. Zhang , Z. Song , Z. Zhou , Y. Deng , W. Zhou , S. Yuan , D. Kou , X. Zhang , Y. Qi , S. Wu , ACS Appl. Mater. Interfaces 2020, 3, 10976.

[7] M. A. Green , E. D. Dunlop , J. Hohl‐Ebinger , M. Yoshita , N. Kopidakis , X. Hao , Prog. Photovoltaics 2022, 30, 3.

[8] T. Gershon , Y. S. Lee , P. Antunez , R. Mankad , S. Singh , D. Bishop , O. Gunawan , M. Hopstaken , R. Haight , Adv. Energy Mater. 2016, 6, 1502468.

[9] W. Ki , H. W. Hillhouse , Adv. Energy Mater. 2011, 1, 732.

[10] L. Guo , J. Shi , Q. Yu , B. Duan , X. Xu , J. Zhou , J. Wu , Y. Li , D. Li , H. Wu , Y. Luo , Q. Meng , Sci. Bull. 2020, 65, 738. 10.1016/j.scib.2020.01.005 36659107

[11] J. Zhou , X. Xu , B. Duan , H. Wu , J. Shi , Y. Luo , D. Li , Q. Meng , Nano Energy 2021, 89, 106405.

[12] Q. Yu , J. Shi , L. Guo , B. Duan , Y. Luo , H. Wu , D. Li , Q. Meng , Nano Energy 2020, 76, 105042.

[13] S. Suresh , A. R. Uhl , Adv. Energy Mater. 2021, 11, 2003743.

[14] S. Ge , H. Gao , R. Hong , J. Li , Y. Mai , X. Lin , G. Yang , ChemSusChem 2019, 12, 1692. 30698923 10.1002/cssc.201803009

[15] J. A. Clark , A. Murray , J.‐m. Lee , T. S. Autrey , A. D. Collord , H. W. Hillhouse , J. Am. Chem. Soc. 2019, 141, 298. 30525570 10.1021/jacs.8b09966

[16] F. Liu , S. Shen , F. Zhou , N. Song , X. Wen , J. A. Stride , K. Sun , C. Yan , X. Hao , J. Mater. Chem. C 2015, 3, 10783.

[17] H. Luan , B. Yao , Y. Li , L. Ruijian , Z. Ding , Y. Sui , Z. Zhang , H. Zhao , L. Zhang , Sol. Energy Mater. Sol. Cells 2019, 195, 55.

[18] A. D. Collord , H. W. Hillhouse , Chem. Mater. 2016, 28, 2067.

[19] C. Niu , Y. Gong , R. Qiu , Q. Zhu , Y. Zhou , S. Hao , W. Yan , W. Huang , H. Xin , J. Mater. Chem. A 2021, 9, 12981.

[20] M. Kumar , A. Dubey , N. Adhikari , S. Venkatesan , Q. Qiao , Energy Environ. Sci. 2015, 8, 3134.

[21] Y.‐P. Lin , T.‐E. Hsieh , Y.‐C. Chen , K.‐P. Huang , Sol. Energy Mater. Sol. Cells 2017, 162, 55.

[22] G. Altamura , L. Grenet , C. Roger , F. Roux , V. Reita , R. Fillon , H. Fournier , S. Perraud , H. Mariette , J. Renew. Sustain. Ener. 2014, 6, 011401.

[23] S.‐Y. Kim , D.‐H. Son , Y.‐I. Kim , S.‐H. Kim , S. Kim , K. Ahn , S.‐J. Sung , D.‐K. Hwang , K.‐J. Yang , J.‐K. Kang , D.‐H. Kim , Nano Energy 2019, 59, 399.

[24] S.‐H. Wu , C.‐W. Chang , H.‐J. Chen , C.‐F. Shih , Y.‐Y. Wang , C.‐C. Li , S.‐W. Chan , Prog. Photovolt.: Res. Appl. 2017, 25, 58.

[25] T. R. Martin , J. K. Katahara , C. N. Bucherl , B. W. Krueger , H. W. Hillhouse , C. K. Luscombe , Chem. Mater. 2016, 28, 135.

[26] Y. Qi , Y. Liu , D. Kou , W. Zhou , Z. Zhou , Q. Tian , S. Yuan , Y. Meng , S. Wu , ACS Appl. Mater. Interfaces 2020, 12, 14213. 32133837 10.1021/acsami.0c02629

[27] J. A. Clark , A. R. Uhl , T. R. Martin , H. W. Hillhouse , Chem. Mater. 2017, 29, 9328.

[28] J. Madarász , P. Bombicz , M. Okuya , S. Kaneko , Solid State Ionics 2001, 141–142, 439.

[29] S. Engberg , F. Martinho , M. Gansukh , A. Protti , R. Kungas , E. Stamate , O. Hansen , S. Canulescu , J. Schou , Sci. Rep. 2020, 10, 20749. 33247169 10.1038/s41598-020-77592-z PMC7699652

[30] J. J. S. Scragg , L. Choubrac , A. Lafond , T. Ericson , C. Platzer‐Björkman , Appl. Phys. Lett. 2014, 104, 041911.

[31] C. J. Hages , M. J. Koeper , C. K. Miskin , K. W. Brew , R. Agrawal , Chem. Mater. 2016, 28, 7703.

[32] P. Zhang , Q. Yu , X. Min , L. Guo , J. Shi , X. Zhao , D. Li , Y. Luo , H. Wu , Q. Meng , S. Wu , RSC Adv. 2018, 8, 4119.

[33] B. Zhang , Y. Lei , R. Qi , H. Yu , X. Yang , T. Cai , Z. Zheng , Sci. China Mater. 2019, 62, 519.

[34] X. Zhai , H. Jia , Y. Zhang , Y. Lei , J. Wei , Y. Gao , J. Chu , W. He , J.‐J. Yin , Z. Zheng , CrystEngComm 2014, 16, 6244.

[35] P. Dong , C. Zhao , Y. Lei , H. Song , S. Wu , Z. Zheng , Cryst. Growth Des. 2021, 21, 4038.

[36] Y. Lei , L. Gu , W. He , Z. Jia , X. Yang , H. Jia , Z. Zheng , J. Mater. Chem. A 2016, 4, 5474.

[37] N. Qu , Y. Lei , X. Yang , X. Hu , W. Zhao , C. Zhao , Z. Zheng , J. Mater. Chem. C 2020, 8, 8451.

[38] L. Guo , M. Liu , Y. Lei , L. Mi , Z. Zheng , CrystEngComm 2021, 23, 2938.

[39] V. Karade , A. Lokhande , P. Babar , M. G. Gang , M. Suryawanshi , P. Patil , J. H. Kim , Sol. Energy Mater. Sol. Cells 2019, 200, 109911.

[40] Y.‐F. Qi , D.‐X. Kou , W.‐H. Zhou , Z.‐J. Zhou , Q.‐W. Tian , Y.‐N. Meng , X.‐S. Liu , Z.‐L. Du , S.‐X. Wu , Energy Environ. Sci. 2017, 10, 2401.

[41] S. G. Haass , M. Diethelm , M. Werner , B. Bissig , Y. E. Romanyuk , A. N. Tiwari , Adv. Energy Mater. 2015, 5, 1500712.

