# Supplementary material for: DMF‐Based Large‐Grain Spanning Cu2ZnSn(S x ,Se1‐ x )4 Device with a PCE of 11.76%
Source: Adv Sci (Weinh). 2022 Apr 28;9(20):2201241. doi: 10.1002/advs.202201241 (PMC9284129; doi:10.1002/advs.202201241)
Supplement: Supplementary file 1 — Supporting Information [file ADVS-9-2201241-s001.pdf]

ADVANCED SCIENCE
Open Access

Supporting Information

for *Adv. Sci.*, DOI 10.1002/advs.202201241

DMF-Based Large-Grain Spanning $Cu_2ZnSn(S_x,Se_{1-x})_4$ Device with a PCE of 11.76%

Yubo Cui, Mengyang Wang, Peizhe Dong, Shuangshuang Zhang, Junjie Fu, Libo Fan, Chaoliang Zhao*, Sixin Wu* and Zhi Zheng**

Supporting Information

DMF-based Large-Grain Spanning $Cu_2ZnSn(S_x,Se_{1-x})_4$ device with a PCE of 11.76%

Yubo Cui, Mengyang Wang, Peizhe Dong, Shuangshuang Zhang, Junjie Fu, Libo Fan, Chaoliang Zhao,* Sixin Wu,* and Zhi Zheng**

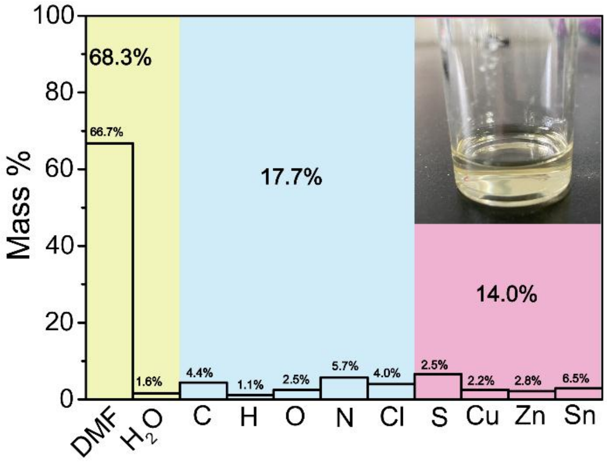

Figure S1. Calculated molar ratio of different substances in the precursor solution, inset is the photograph of the precursor solution.

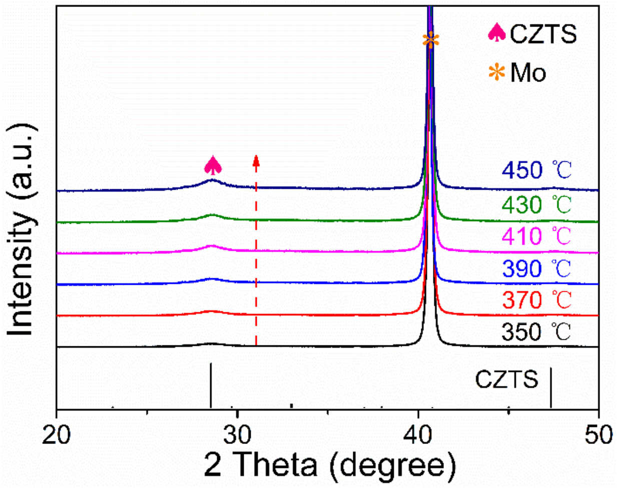

Figure S2. XRD patterns of precursor thin films at different pre-annealing temperatures.

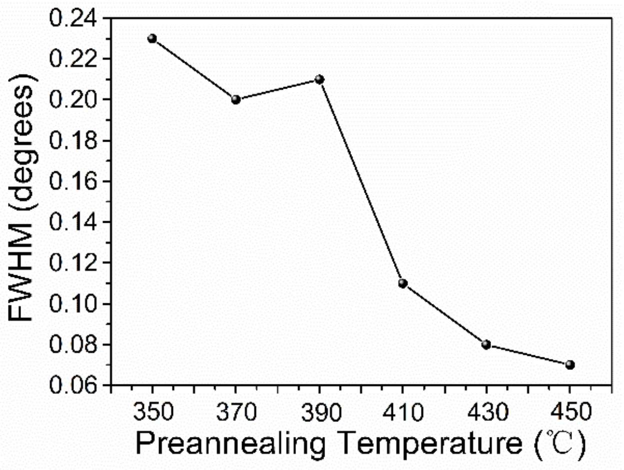

Figure S3. FWHM of (112) crystalline plane in XRD patterns of CZTSSe prepared at different pre-annealing temperatures.

Table S1. $I_{(112)}/I_{(204)}$ values of the CZTSSe films.

Pre-annealing Tempurature (℃)	$I_{(112)}/I_{(204)}$
350	2.39
370	2.85
390	2.69
410	3.10
430	3.07
450	3.81

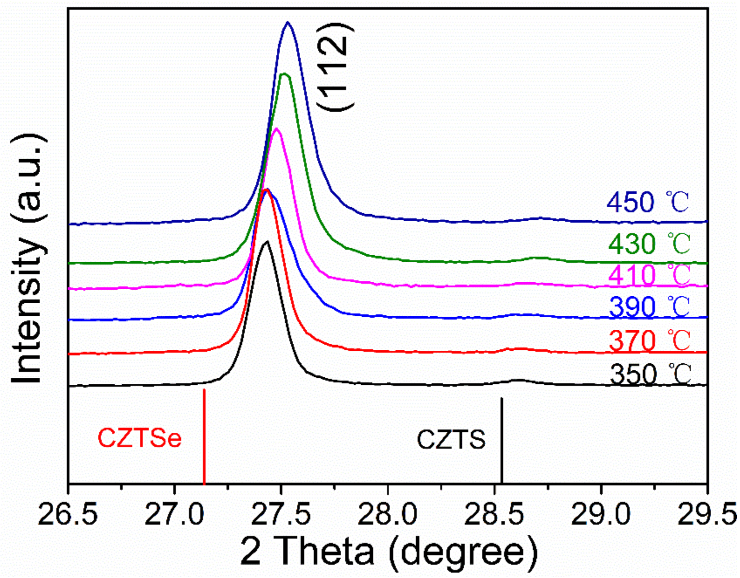

Figure S4. XRD patterns corresponding to the (111) plane diffraction peak at varied temperature.

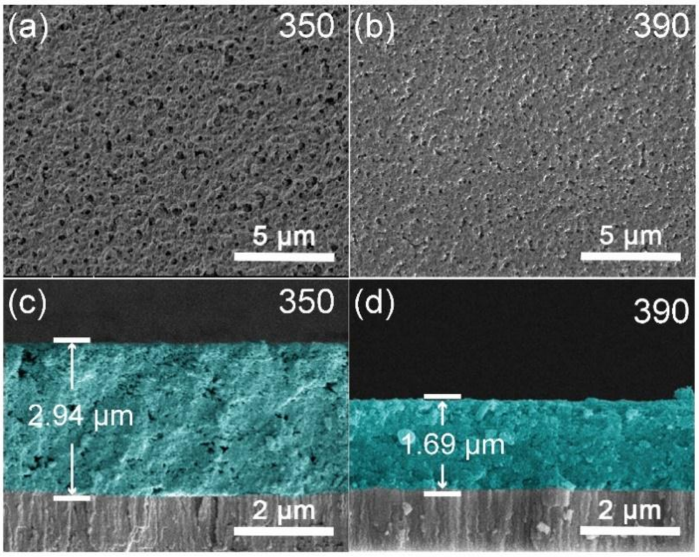

Figure S5. SEM images of Surface and cross-sectional of the precursor films prepared at different pre-annealing temperature (a) 350°C surface , (b) 390°C surface, (c) 350°C cross-section, (d) 390°C cross-section.

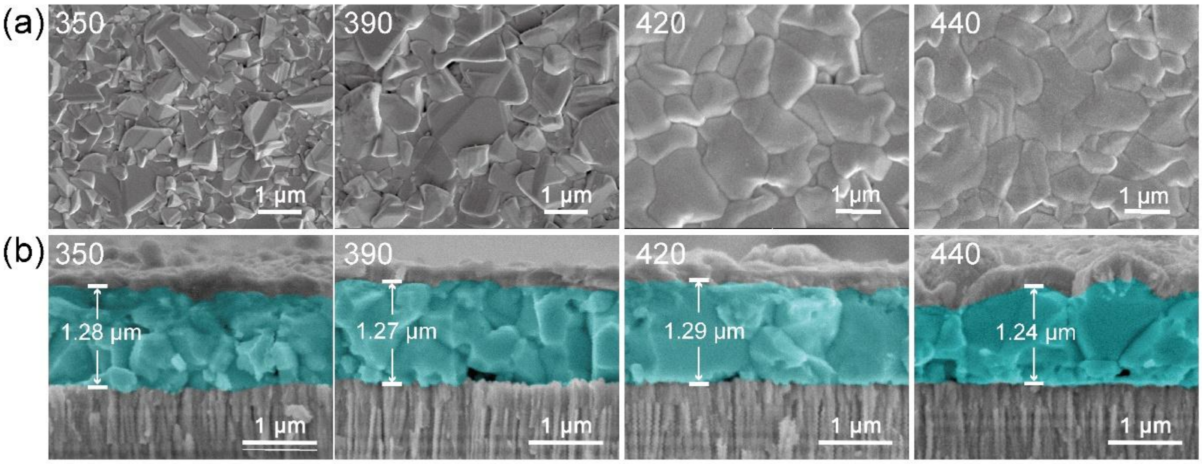

Figure S6. SEM images of the surface and cross-section of the absorbers prepared at different pre-annealing temperature (a) surface at 350°C, 390°C, 420°C, and 440°C, (b) cross-section at 350°C, 390°C, 420°C, and 440°C.

The PCE of films at 420°C and 440 °C are 11.33% and 10.29%, respectively.

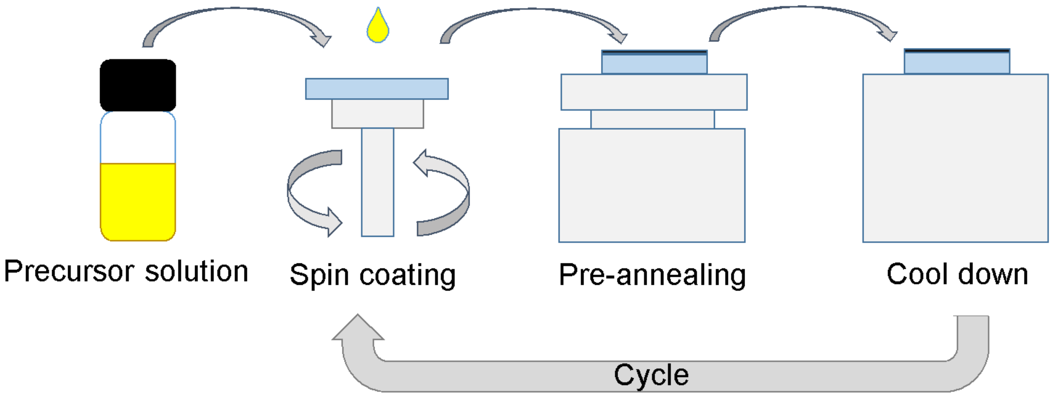

Figure S7. Preparation of precursor films with different spin-coating layers.

To analyze the crystallization of CZTS at different depths of the precursor films and the degree of graphitization of the polymer, spin-coated film samples with varied spin-coating layers of 1~6 were prepared. Preparation of precursor films with different spin-coating layers in **Figure 3** were shown in Figure S7, which including spin coating, pre-annealing, and cool down. To be noticed, samples with 1~6 spin-coating layers went through same 6 times of pre-annealing. For instance, 1 layer only went through spin-coating once but 6 times of pre-annealing, 2 layers was prepared by 2 times of spin-coting and 6 times of pre-annealing. Similarly, Samples of 3~6 spin-coating layers were fabricated. Finally, samples with 1~6 spin-coating layers at pre-annealing temperatures of 370 ℃, 430 ℃ and 450 ℃ were obtained respectively.

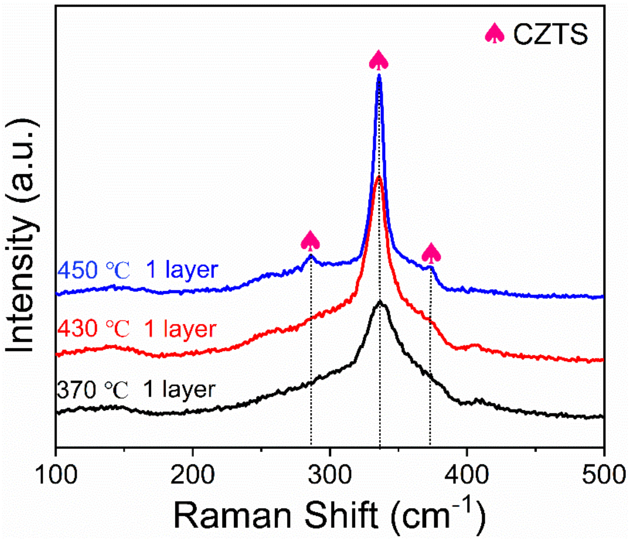

Figure S8. Raman spectra of 1 spin-coating layer samples pre-annealed at 370 ℃, 430 ℃ and 450 ℃ in range of 100~500 cm^{-1}.

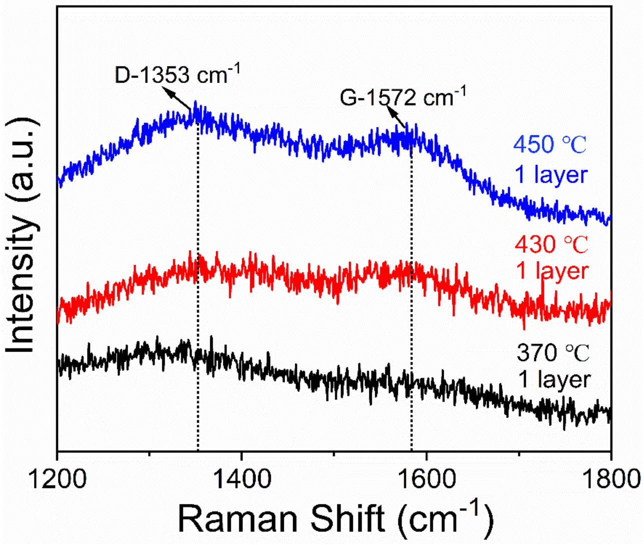

Figure S9. Raman spectra of 1 spin-coating layer samples pre-annealed at 370 ℃, 430 ℃ and 450 ℃ in range of 1200~1800 cm^{-1}.

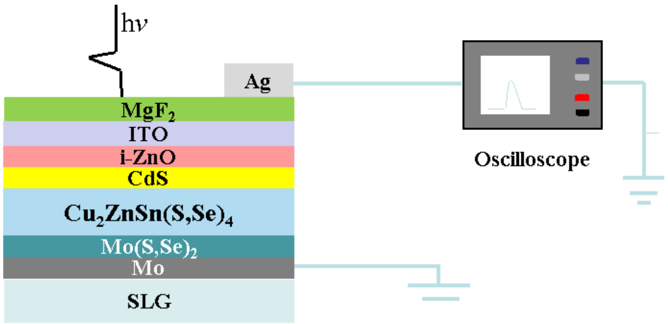

Figure S10. TSPV test configuration of CZTSSe solar cells.

Figure S10 was TSPV test configuration of CZTSSe solar cells. 355 nm pulse laser with a pulse width of 4 ns was first processed by a reflector and then utilized for exciting the CZTSSe solar cells without amplifier. The signals were recorded by a digital TDS oscilloscope.

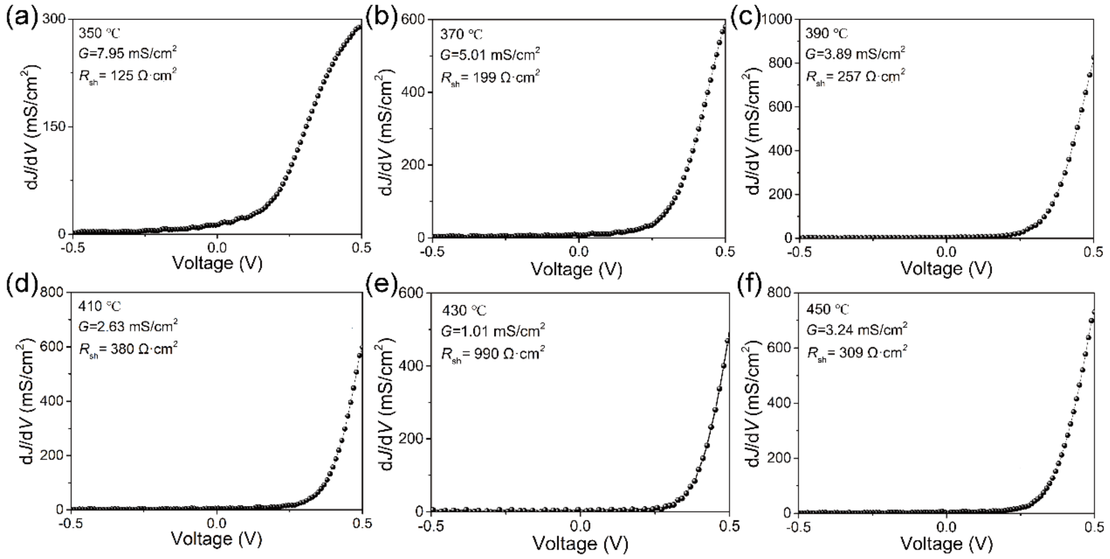

Figure S11. d*J*/d*V* vs *V* curves of CZTSSe samples prepared at different pre-annealing temperature deduced from J−V curves with standard light ; G is the average value of the reverse bias region of the curve, $R_{sh}=1/G$, (a) 350℃, (b) 370℃, (c) 390℃, (d) 410℃, (e) 430℃, (f) 450℃.

Table S2. Parameters of devices prepared at different pre-annealing temperature

Pre-temperature (℃)	PCE (%)	V_{oc} (mV)	J_{sc} (mA/cm^2)	FF (%)	R_{sh} (Ω·cm^2)	R_s (Ω·cm^2)	G (mS/cm^2)	A
350	6.89	419	28.92	56.9	125	0.53	7.95	1.85
370	8.36	445	29.95	62.8	199	0.48	5.01	1.46
390	9.86	474	32.81	63.4	257	0.35	3.89	1.49
410	11.09	491	35.02	64.6	380	0.38	2.63	1.35
430	11.76	501	35.36	66.4	990	0.53	1.01	1.25
450	9.23	460	32.22	62.7	309	0.47	3.24	1.43

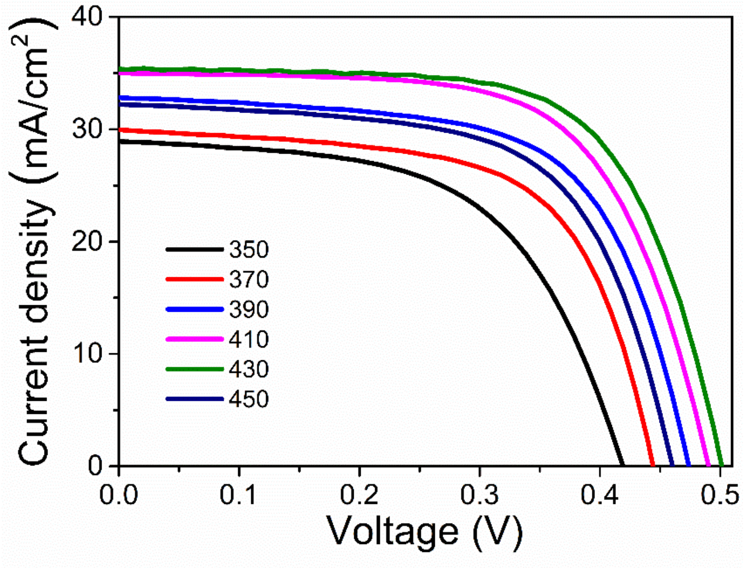

Figure S12. JV curves of devices prepared at different pre-annealing temperature

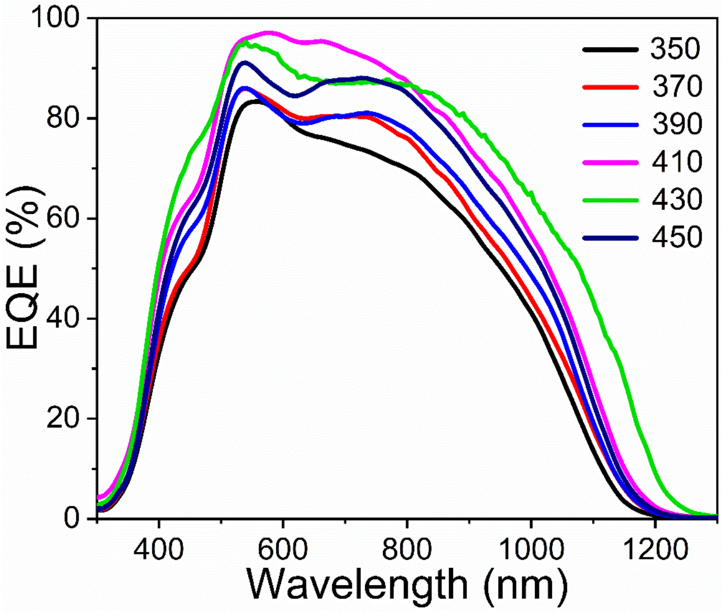

Figure S13. EQE curves of devices prepared at different pre-annealing temperature
